# Association of Vortex Vein Morphology with Punctate Hyperfluorescence on ICGA in Neovascular Age-Related Macular Degeneration

**DOI:** 10.3390/jcm15155882

**Published:** 2026-07-28

**Authors:** Hiroyuki Kamao, Katsutoshi Goto, Kenichi Mizukawa, Ryutaro Hiraki, Atsushi Miki, Shuhei Kimura

**Affiliations:** 1 Department of Ophthalmology, Kawasaki Medical School, 577 Matsushima, Kurashiki 701-0192, Japan; 2 Department of Ophthalmology, Shirai Eye Hospital, 1339 Takasecho, Kamitakase, Mitoyo 767-0001, Japan

**Keywords:** punctate hyperfluorescence, indocyanine green angiography, vortex vein morphology, watershed zone, neovascular age-related macular degeneration

## Abstract

**Background/Objectives**: Punctate hyperfluorescence (PH) on indocyanine green angiography (ICGA) is frequently observed in pachychoroid disease. However, the spatial relationship between PH lesions and vortex vein abnormalities remains unclear. This study quantified PH distribution in fellow eyes of patients with unilateral neovascular age-related macular degeneration (nAMD) and examined its association with vortex vein asymmetry and watershed-zone status. **Methods:** This retrospective observational study included 58 fellow eyes with PH from patients with unilateral nAMD. PH lesions were quantified on late-phase ICGA images using ImageJ software. Vortex vein distribution was classified as symmetric, superior-dominant, or inferior-dominant. Eyes were also classified by the presence of a horizontal watershed zone. PH distribution was compared by vortex vein morphology. **Results:** A watershed zone was absent in 14 eyes (24.1%). Vortex vein distribution was asymmetric in 27 eyes (46.6%), including 17 (29.3%) superior-dominant and 10 (17.2%) inferior-dominant. Overall, 3401 PH lesions were detected. Eyes without a watershed zone had significantly more PH lesions than those with a watershed zone (*p* = 0.002). The proportion of superior-region PH lesions differed significantly by vertical vortex vein predominance, with preferential distribution on the dominant side (*p* < 0.001). Although PH distribution did not differ significantly among the four quadrants overall, 30 eyes (51.7%) had >50% of PH lesions clustered in a single quadrant. **Conclusions:** PH lesions were preferentially distributed on the dominant side of vortex vein asymmetry, and eyes without a watershed zone had more PH lesions. These findings indicate that PH distribution and lesion burden are associated with vortex vein morphology and watershed-zone status and may reflect underlying alterations in choroidal venous drainage.

## 1. Introduction

Neovascular age-related macular degeneration (nAMD) is a leading cause of blindness in developed countries. In addition to conventional drusen-associated nAMD, nAMD arising from a pachychoroid background has been increasingly recognized [1]. Pachychoroid refers to a choroidal phenotype characterized by choroidal thickening, dilated outer choroidal vessels known as pachyvessels, and choroidal vascular hyperpermeability (CVH) [2,3]. The pachychoroid disease spectrum includes pachychoroid pigment epitheliopathy (PPE) [2], central serous chorioretinopathy (CSC) [4], pachychoroid neovasculopathy (PNV) [5], and polypoidal choroidal vasculopathy (PCV) [6]. Choroidal structural evaluations using ultra-widefield indocyanine green angiography (ICGA) [7] and en face swept-source optical coherence tomography (OCT) [8] have shown that vortex vein congestion may play an important role in pachychoroid disease pathophysiology. Eyes with pachychoroid disease frequently show asymmetric distribution of the superior and inferior vortex veins and marked dilation of the dominant vortex veins [9]. Choroidal vascular dilation is also associated with CVH and vortex vein congestion [10]. Vortex vein anastomoses, which may develop as a compensatory mechanism to reduce venous congestion, have also been reported [11]. These findings suggest that dilation of the outer choroidal vessels in pachychoroid disease may occur secondary to vortex vein congestion, a mechanism described as venous overload choroidopathy [12]. Thus, pachychoroid diseases appear to share common choroidal vascular abnormalities.

Punctate hyperfluorescence (PH) is an ICGA finding observed across the pachychoroid disease spectrum. PH appears as hyperfluorescent spots during the mid-to-late phases of ICGA and has been reported in affected eyes with CSC [13] and PCV [14] as well as in their fellow eyes. PH may also be associated with the response to anti-vascular endothelial growth factor (VEGF) therapy in nAMD. For example, eyes with PH showed a lower retreatment rate after the loading phase of intravitreal aflibercept therapy [15]. Among eyes with aflibercept-refractory nAMD switched to intravitreal brolucizumab, complete retinal fluid resolution was more frequent in eyes with PH than in those without PH [16]. These findings are consistent with reports that eyes with PNV respond more favorably to anti-VEGF therapy than non-PNV eyes [17,18]. Although anti-VEGF therapy has substantially improved the visual prognosis of nAMD, continuous treatment is required to maintain favorable outcomes, imposing economic and time burdens on patients. Therefore, identifying factors associated with anti-VEGF treatment response may help optimize individualized treatment regimens. PH may serve as an imaging biomarker of treatment responsiveness, but its pathophysiological significance remains unclear.

Although PH lesions frequently occur within areas of CVH [13], histological evidence is lacking, and their anatomical substrate and formation mechanism remain unclear. In addition, the spatial relationship between PH lesions and asymmetry in vortex vein distribution, a key feature of pachychoroid disease pathophysiology, has not been clarified. Clarifying the relationship between PH distribution and vortex vein morphology may provide insight into PH pathogenesis.

Therefore, we quantified PH lesion distribution and examined whether PH lesions preferentially localize to regions of vortex vein predominance or altered choroidal drainage, as indicated by vortex vein asymmetry and watershed-zone status. We aimed to clarify whether PH distribution is associated with choroidal vascular morphology within the pachychoroid disease spectrum.

## 2. Materials and Methods

### 2.1. Study Design

This retrospective observational study was conducted at Kawasaki Medical School. We reviewed the medical charts and imaging data of consecutive treatment-naïve patients with unilateral nAMD treated at our hospital between January 2020 and September 2025. Unilateral nAMD was defined as the presence of active macular neovascularization (MNV) in one eye and the absence of MNV findings in the fellow eye. The fellow eye was considered non-MNV when all of the following were absent: retinal or subretinal hemorrhage on fundus examination, intraretinal or subretinal fluid and flat irregular pigment epithelium detachment on OCT, leakage on fluorescein angiography (FA), and an abnormal vascular network on ICGA. Demographic data and systemic comorbidities, including hypertension, diabetes mellitus, and smoking status, were extracted from medical records. Smoking status was classified as never-smoker or ever-smoker according to a previously published classification [15]. The exclusion criteria were prior laser photocoagulation or vitrectomy; high myopia (≥6.0 diopters), uveitis, or angioid streaks; and other ocular diseases that could affect outcomes in the study eye, including branch retinal vein occlusion, diabetic retinopathy, and glaucoma.

### 2.2. Data Collection

Both fellow and affected eyes underwent indirect ophthalmoscopy, slit-lamp biomicroscopy with a non-contact lens, color fundus photography, swept-source OCT, FA, and ICGA. Swept-source OCT was performed using either the DRI OCT-1 Atlantis (Topcon Corporation, Tokyo, Japan) or the Xephilio OCT-S1 (Canon Inc., Tokyo, Japan). For vortex vein and watershed-zone assessments, en face OCT images obtained using the DRI OCT-1 Atlantis were evaluated in 51 eyes, and those obtained using the Xephilio OCT-S1 were evaluated in 7 eyes. FA and ICGA were performed using both the HRA-2 (Heidelberg Engineering GmbH, Dossenheim, Germany) and Optos California (Optos plc, Dunfermline, UK). DRI OCT-1 Atlantis line scans were obtained in all eyes and used to measure subfoveal choroidal thickness (SFCT) according to previously published methods [15]. A 12 × 9 mm macula-centered swept-source OCT scan was obtained to generate en face OCT images flattened with reference to Bruch’s membrane. Choroidal segmentation was performed automatically and manually corrected when necessary. After segmentation between Bruch’s membrane and the choroid–sclera interface, a 100 µm thick slab centered at the level corresponding to 50% of the total SFCT from Bruch’s membrane was selected. En face OCT images were then reconstructed from this choroidal slab for vortex vein evaluation.

### 2.3. Image Analysis

**Figure 1:**
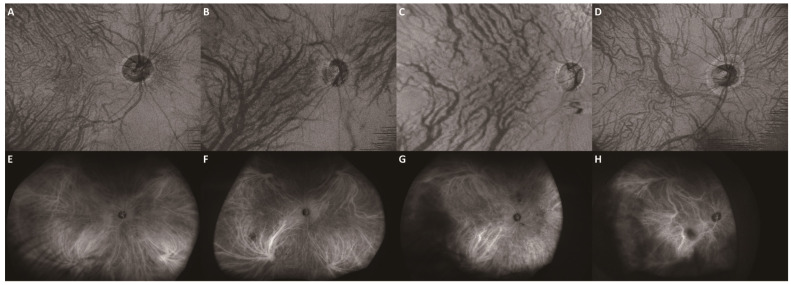
Classification of vortex vein distribution. Representative en face OCT images (**A**–**D**) and ICGA images (**E**–**H**) show vortex vein distribution according to the presence or absence of a horizontal watershed zone and vortex vein symmetry. Eyes were classified as having symmetric or asymmetric vortex veins according to whether the boundary line passed through the optic disc. Representative images show eyes with a watershed zone and symmetric vortex veins (**A**,**E**), eyes with a watershed zone and asymmetric vortex veins with inferior-dominant distribution (**B**,**F**), eyes without a watershed zone and symmetric vortex veins (**C**,**G**), and eyes without a watershed zone and asymmetric vortex veins with superior-dominant distribution (**D**,**H**).

PH was defined according to our previous report [15]. PH was identified in the mid-to-late phases of ICGA images acquired with the HRA-2 as solitary or clustered hyperfluorescent spots typically distributed along choroidal vessels within areas of CVH. Watershed-zone assessment focused on the horizontal watershed zone crossing the macular area. Eyes were classified according to the presence or absence of this watershed zone on ICGA and en face OCT images. Eyes without a watershed zone were defined as those in which the boundary between the superior and inferior vortex vein territories was indistinct because of vortex vein anastomoses. A boundary line was defined to classify vortex vein distribution as symmetric or asymmetric. In eyes with a watershed zone, the boundary line was drawn along the center of the watershed zone. In eyes without a watershed zone, the boundary line was estimated from the course and curvature of the choroidal vessels as the presumed junction between the superior and inferior vortex vein territories. Vortex vein distribution was classified as symmetric when the boundary line intersected the optic disc and asymmetric when it did not. In asymmetric eyes, vertical predominance was further classified as superior-dominant or inferior-dominant according to whether the superior or inferior vortex vein system was more prominent (Figure 1). Two masked retinal specialists (H.K. and K.G.) independently assessed the presence or absence of the watershed zone and vortex vein distribution. Discrepancies were adjudicated by a senior grader (K.M.).

**Figure 2:**
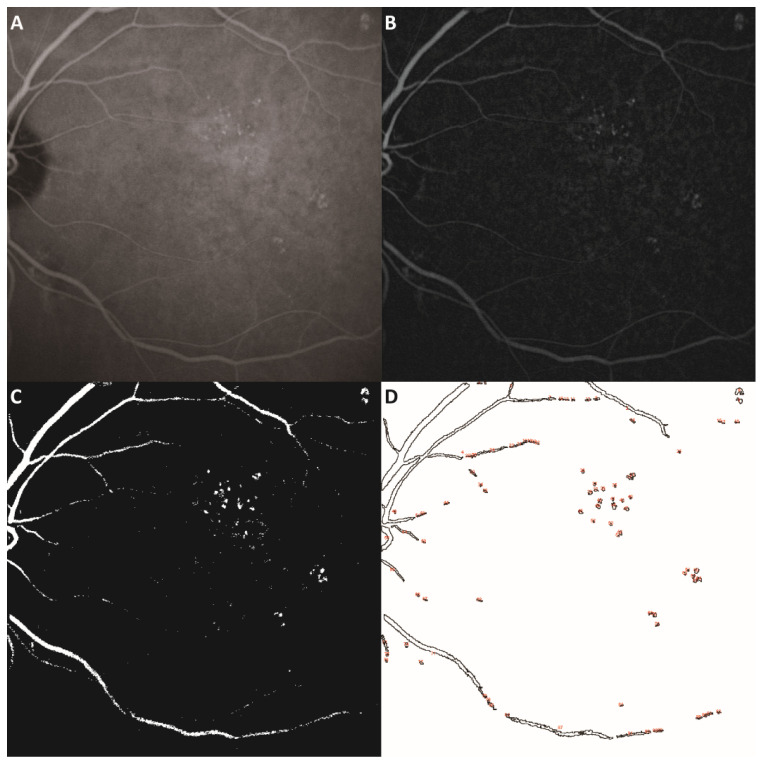
Quantitative analysis of PH lesions. (**A**) Late-phase ICGA image showing clustered PH lesions within an area of CVH in the macular region. (**B**) After conversion to an 8-bit grayscale image and background correction, CVH, PH lesions, and retinal vessels were visualized as hyperfluorescent signals. (**C**) The image was binarized using a cutoff value defined as the mean brightness plus 6 standard deviations, calculated from four background regions of interest. (**D**) Hyperfluorescent signals were automatically detected using the Analyze Particles function. The detected signals included both PH lesions and retinal vessels. PH, punctate hyperfluorescence; ICGA, indocyanine green angiography; CVH, choroidal vascular hyperpermeability.

**Figure 3:**
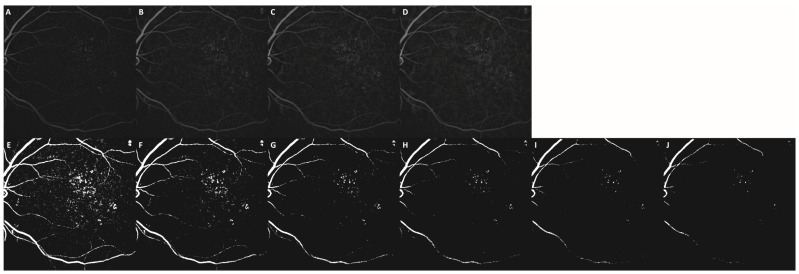
Comparison of candidate parameters for PH lesion detection. Background-corrected late-phase ICGA images obtained using rolling-ball radii of 10 (**A**), 20 (**B**), 30 (**C**), and 40 pixels (**D**). Binarized images obtained using cutoff values of the mean background brightness plus 3 (**E**), 4 (**F**), 5 (**G**), 6 (**H**), 7 (**I**), and 8 standard deviations (**J**).

PH lesions were quantitatively analyzed using ImageJ software (version 1.54p; National Institutes of Health, Bethesda, MD, USA). Late-phase ICGA images acquired with the HRA-2 at least 13 min after dye injection were used for analysis (Figure 2A). Images were converted to 8-bit grayscale, and background correction was performed using the Subtract Background function with a rolling-ball radius of 20 pixels (Figure 2B). Four 20 × 20-pixel background regions of interest were placed near the retinal arcade vessels in the superotemporal, inferotemporal, superonasal, and inferonasal quadrants; the mean background brightness plus 6 standard deviations was then used as the cutoff value for binarization (Figure 2C). Because no previously validated parameters were available for quantitative PH assessment, candidate settings were empirically compared in 20 randomly selected eyes. Rolling-ball radii of 10, 20, 30, and 40 pixels (Figure 3A–D) and binarization cutoffs ranging from the mean background brightness plus 3 to 8 standard deviations (Figure 3E–J) were reviewed by three retinal specialists (H.K., K.G., and K.M.). By consensus, a rolling-ball radius of 20 pixels and a cutoff of the mean background brightness plus 6 standard deviations were selected because these settings provided the most appropriate balance between background suppression and preservation of discrete PH lesions while minimizing false-positive noise and lesion loss. These fixed parameters were subsequently applied uniformly to all study images. PH candidates were then automatically detected using the Analyze Particles function (Figure 2D). After manual exclusion of hyperfluorescent signals originating from retinal vessels, the remaining lesions were defined as PH lesions, and their size and location were assessed quantitatively. PH lesion clustering was operationally defined at the eye level as >50% of the total PH lesions in an individual eye being located in a single quadrant. Sensitivity analyses were performed using alternative thresholds of >40% and >60%. To assess intergrader reproducibility, two graders (H.K. and K.G.) independently classified all automatically detected candidate lesions. To assess intragrader reproducibility, H.K. repeated the classification in 20 randomly selected eyes after an interval of at least two weeks while masked to the initial classifications. The size of each PH lesion was classified according to conventional soft drusen size categories: small, <63 μm; medium, ≥63 to <125 μm; and large, ≥125 μm. PH size was classified using pixel-based thresholds determined from the diameter of a major retinal vein crossing the optic disc margin, which served as an internal reference scale. In 10 representative eyes in which a major retinal vein crossing the optic disc margin was fully visible and could be measured reliably, the mean vessel diameter was approximately 14 pixels, corresponding to approximately 125 μm. Therefore, PH lesions with diameters of ≥7 and ≥14 pixels were classified as medium and large PH, respectively. These thresholds corresponded to estimated lesion areas of approximately 38.5 and 153.9 pixels^2^, respectively. These operational thresholds were adapted from conventional soft drusen size categories for descriptive purposes and do not imply anatomical and biological equivalence to drusen.

### 2.4. Statistical Analysis

Statistical analyses were performed using JMP Student Edition, version 19.1.3 (JMP Statistical Discovery LLC, Cary, NC, USA). Continuous variables were presented as mean ± standard deviation or median and interquartile range, as appropriate, and categorical variables were expressed as number and percentages. The main analysis was the proportion of PH lesions located in the superior region in each eye. The main comparison was the overall difference in this proportion among eyes with inferior-dominant, non-dominant, and superior-dominant vortex vein distributions, assessed using the Kruskal–Wallis test. Pairwise comparisons were conducted as post hoc analyses using the Steel–Dwass procedure, with multiplicity-adjusted *p* values and Hodges–Lehmann location-shift estimates with 95% confidence intervals reported for each comparison. All other analyses, including those involving watershed-zone status, total and size-specific PH lesion number, clinical characteristics, and quadrant-level distribution, were considered secondary or exploratory. Baseline characteristics of the included and excluded eyes were summarized descriptively. Between-group differences were reported as differences in means or proportions with 95% CIs, and absolute standardized differences were calculated. Differences in categorical variables were expressed as percentage-point differences. The association between watershed-zone status and vortex vein distribution was evaluated using Fisher’s exact test. Associations of total PH lesion number with clinical and imaging variables were evaluated using univariable negative binomial regression, and incidence rate ratios (IRRs) with 95% CIs were calculated. Because PH lesion numbers were overdispersed, negative binomial regression was selected instead of Poisson regression. Associations of continuous clinical variables with total PH lesion number were also evaluated using Spearman’s rank correlation coefficients. The association between watershed-zone status and total PH lesion number was further evaluated using multivariable negative binomial regression adjusted for age, sex, SFCT, and soft-drusen status. The dispersion parameter and model-fit indices, including the corrected Akaike information criterion, Bayesian information criterion, and generalized *R*^2^, were reported. Because only nine eyes had soft drusen, a sensitivity analysis was also performed using a model excluding soft-drusen status. The distribution of PH lesions among the four quadrants was assessed using the Friedman test. Intergrader agreement for the assessment of watershed-zone status and vortex vein distribution was evaluated using Cohen’s kappa coefficient with 95% CIs. Intergrader and intragrader agreement for candidate classification was evaluated using percentage agreement and Cohen’s kappa coefficient with 95% CIs. Eye-level intergrader agreement for the total PH lesion number and the proportion of PH lesions located in the superior region was assessed using intraclass correlation coefficients (ICCs) based on a two-way mixed-effects model for absolute agreement with single measurements, together with Bland–Altman analyses. No formal multiplicity adjustment was applied across the secondary and exploratory analyses, which were interpreted as hypothesis-generating; however, multiplicity-adjusted *p* values were used for the Steel–Dwass post hoc comparisons. Statistical significance was set at *p* < 0.05.

## 3. Results

### 3.1. Demographic and Clinical Characteristics of the Study Population

**Figure 4:**
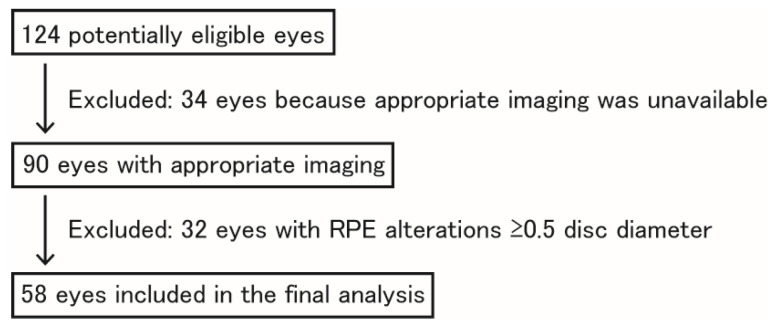
Flow diagram of participant selection. RPE, retinal pigment epithelium.

A total of 124 fellow eyes with PH from patients with unilateral nAMD were initially reviewed. Of these, 34 eyes were excluded because late-phase ICGA or en face OCT images were unavailable. Among the remaining 90 eyes, 32 were further excluded because they showed retinal pigment epithelium (RPE) alterations extending over an area of ≥0.5 disc diameter. Consequently, 58 eyes were included in the final analysis (Figure 4). Baseline characteristics of the included and excluded eyes are presented in Table 1. The absolute standardized differences were relatively small for age, sex, diabetes, smoking history, soft drusen, and choroidal thickness. In contrast, hypertension showed the largest between-group imbalance and was more prevalent in the included group than in the excluded group.

### 3.2. Morphology of Vortex Veins

A watershed zone was observed in 44 eyes (75.9%) and absent in 14 eyes (24.1%). Vortex vein distribution was symmetric in 31 eyes (53.4%) and asymmetric in 27 eyes (46.6%), including superior-dominant distribution in 17 eyes (29.3%) and inferior-dominant distribution in 10 eyes (17.2%). There was no significant difference in the frequencies of superior- and inferior-dominant patterns (*p* = 0.18). Intergrader agreement was excellent for both watershed-zone status and vortex vein distribution, with κ coefficients of 0.91 and 0.91, respectively. Among eyes with a watershed zone, vortex vein distribution was symmetric in 24 eyes (54.5%), superior-dominant in 12 eyes (27.3%), and inferior-dominant in 8 eyes (18.2%). Among eyes without a watershed zone, vortex vein distribution was symmetric in 7 eyes (50.0%), superior-dominant in 5 eyes (35.7%), and inferior-dominant in 2 eyes (14.3%). Vortex vein distribution did not differ significantly between eyes with and without a watershed zone (*p* = 0.84). Hypertension was present in 26 of 44 eyes (59.1%) with a watershed zone and in 8 of 14 eyes (57.1%) without a watershed zone, with no significant association between hypertension and watershed-zone status (*p* = 1.00). The prevalence of hypertension was 40.0% in eyes with inferior-dominant vortex veins, 61.3% in eyes with symmetric vortex veins, and 64.7% in eyes with superior-dominant vortex veins, and did not differ significantly among the three groups (*p* = 0.41).

### 3.3. Characteristics and Distribution of PH

Among 5539 automatically detected candidate lesions in the 58 eyes, the two graders agreed on the classification of 5196 candidates (93.8%), with a Cohen’s κ of 0.87 (95% CI, 0.86–0.89), indicating excellent intergrader agreement. The proportions of candidates classified as PH lesions were 61.4% by H.K. and 62.2% by K.G. The corresponding ICC for the total PH lesion number was 0.997. At the eye level, Bland–Altman analysis showed a mean intergrader difference of 0.88 lesions for the total PH lesion number, with 95% limits of agreement from −10.9 to 12.7 lesions. Intragrader reproducibility for H.K. was evaluated in a subset of 20 eyes comprising 1888 automatically detected candidate lesions. H.K. agreed with the initial classification for 1820 candidates (96.4%), with a Cohen’s κ of 0.93 (95% CI, 0.91–0.94), indicating excellent intragrader agreement. Among the 58 eyes included in the final analysis, 3401 PH lesions were detected by image analysis and included in the lesion-based analysis. Each PH lesion was classified by size according to conventional soft drusen size categories. Of the 3401 PH lesions, 2881 (84.7%) were classified as small, 490 (14.4%) as medium-sized, and 30 lesions (0.9%) as large. In the analysis of factors associated with total PH lesion number, older age was significantly associated with a higher total PH lesion number (IRR per 1-year increase, 1.04; 95% CI, 1.02–1.07; *p* = 0.001). The presence of soft drusen in the fellow eye was significantly associated with a lower total PH lesion number (IRR, 0.53; 95% CI, 0.29–0.97; *p* = 0.04). By contrast, sex, smoking history, hypertension, diabetes mellitus, presence of polypoidal lesion in the affected eye and subfoveal choroidal thickness were not significantly associated with total PH lesion number (Table 2).

**Figure 5:**
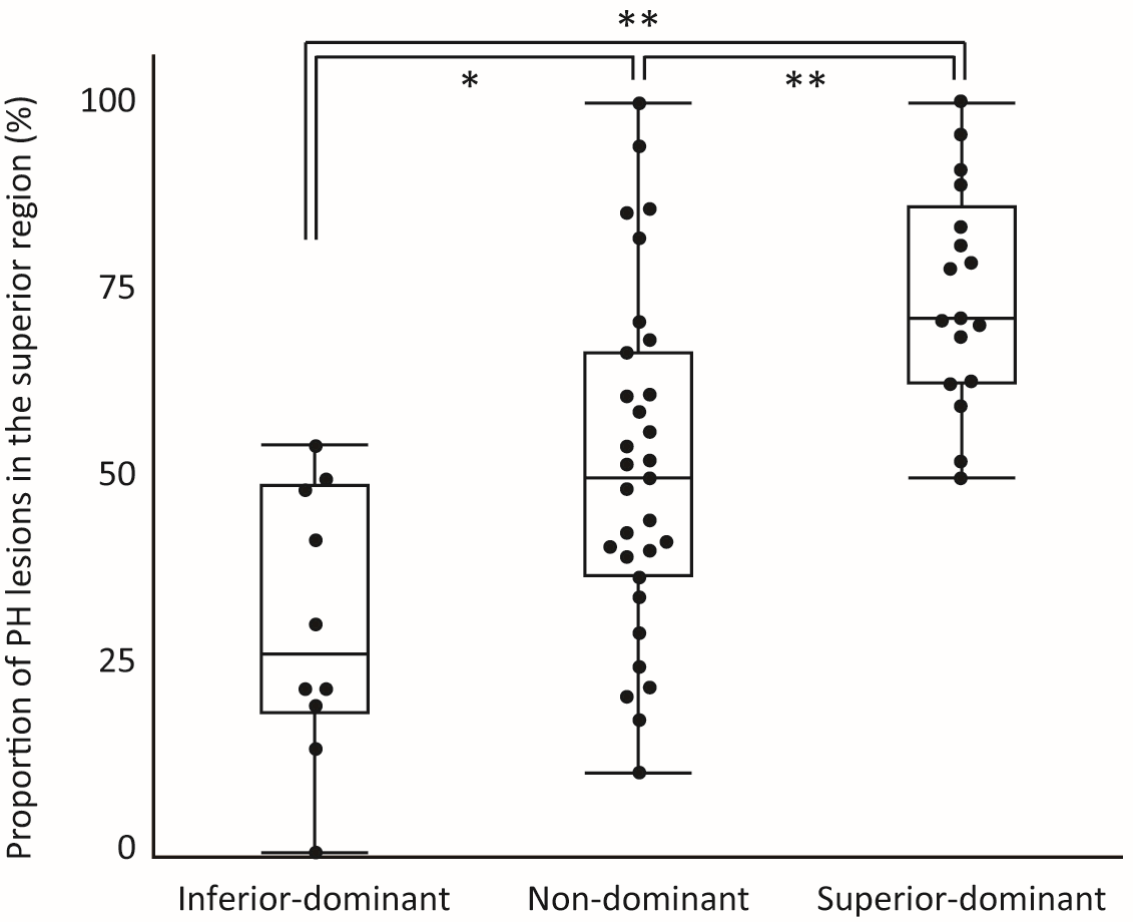
Proportion of PH lesions located in the superior region according to vortex vein distribution. Boxes indicate the median and interquartile range, whiskers indicate the minimum and maximum values, and individual dots represent eyes. Pairwise *p* values were multiplicity-adjusted using the Steel–Dwass procedure. * *p* < 0.05, ** *p* < 0.01. PH, punctate hyperfluorescence.

In the vertical distribution analysis, 1885 PH lesions were located in the superior region and 1516 in the inferior region. Total PH lesion number was compared between eyes with and without a watershed zone. Eyes without a watershed zone had significantly more PH lesions than those with a watershed zone (median (IQR), 67.5 (28.8–103.5) vs. 32.0 (18.0–59.8) lesions). In univariable negative binomial regression, the absence of a watershed zone was significantly associated with a higher total PH lesion number (IRR, 2.28; 95% CI, 1.41–3.69; *p* < 0.001). In multivariable negative binomial regression adjusted for age, sex, SFCT, and soft-drusen status, older age (adjusted IRR per year, 1.05; 95% CI, 1.02–1.07; *p* < 0.001) and the absence of a watershed zone (adjusted IRR, 2.01; 95% CI, 1.28–3.14; *p* = 0.002) remained significantly associated with a higher total PH lesion number, whereas the presence of soft drusen was associated with a lower total PH lesion number (adjusted IRR, 0.50; 95% CI, 0.29–0.87; *p* = 0.014). The estimated dispersion parameter for the multivariable negative binomial model was 0.464 (95% CI, 0.298–0.629). The model yielded an AICc of 571.86, a BIC of 584.05, and a generalized *R*^2^ of 0.412. In a sensitivity analysis excluding soft-drusen status, the absence of a watershed zone remained significantly associated with a higher total PH lesion number after adjustment for age, sex, and SFCT (adjusted IRR, 2.27; 95% CI, 1.45–3.57; *p* < 0.001). Because total PH lesion number varied among eyes, the proportions of PH lesions in the superior and inferior regions were calculated for each eye and then averaged across all eyes. The mean proportions of PH lesions were 54.5 ± 24.6% in the superior region and 45.5 ± 24.6% in the inferior region. To determine whether PH lesion distribution was associated with vertical vortex vein predominance, the proportion of superior-region PH lesions was compared among eyes with inferior-dominant, non-dominant, and superior-dominant vortex vein distributions. The median proportion of superior-region PH lesions was 26.5% (IQR, 18.6–48.9%) in eyes with inferior-dominant vortex veins, 50.0% (IQR, 36.8–66.7%) in eyes with non-dominant vortex veins, and 71.2% (IQR, 62.7–86.1%) in eyes with superior-dominant vortex veins. The proportion of superior-region PH lesions differed significantly among the three groups (*p* < 0.001). Multiplicity-adjusted post hoc comparisons using the Steel–Dwass procedure showed significant differences between superior-dominant and non-dominant eyes (Hodges–Lehmann location-shift estimate, 24.3 percentage points; 95% CI, 9.4–38.9; adjusted *p* = 0.002) and between superior-dominant and inferior-dominant eyes (44.1 percentage points; 95% CI, 26.4–62.5; adjusted *p* < 0.001). The difference between non-dominant and inferior-dominant eyes was of borderline significance (19.7 percentage points; 95% CI, 0.00–39.9; adjusted *p* = 0.045) (Figure 5). Eye-level intergrader agreement for the proportion of PH lesions located in the superior region was excellent, with an ICC of 0.937. Bland–Altman analysis showed a mean intergrader difference of −0.59 percentage points, with 95% limits of agreement from −17.29 to 16.12 percentage points.

In the quadrant analysis, 1030 PH lesions were located in the superotemporal quadrant, 735 in the inferotemporal quadrant, 855 in the superonasal quadrant, and 781 in the inferonasal quadrant. The median proportions of PH lesions in each quadrant were 27.2% (IQR, 9.4–44.5%) in the superotemporal quadrant, 18.7% (IQR, 9.0–33.3%) in the inferotemporal quadrant, 21.7% (IQR, 9.7–37.5%) in the superonasal quadrant, and 16.7% (IQR, 6.0–29.3%) in the inferonasal quadrant. The proportion of PH lesions did not differ significantly among the four quadrants (*p* = 0.27). PH lesion clustering, defined as >50% of PH lesions being located in a single quadrant, was observed in 30 eyes (51.7%). The clustered quadrant was superotemporal in 12 eyes, inferotemporal in 3 eyes, superonasal in 6 eyes, and inferonasal in 9 eyes. In sensitivity analyses, at least one quadrant contained >40% and >60% of the total PH lesions in 41 eyes (70.7%) and 19 eyes (32.8%), respectively. At the >40% threshold, the qualifying quadrant was superotemporal in 16 eyes, inferotemporal in 7 eyes, superonasal in 9 eyes, and inferonasal in 11 eyes. At the >60% threshold, the qualifying quadrant was superotemporal in 5 eyes, inferotemporal in 3 eyes, superonasal in 4 eyes, and inferonasal in 7 eyes. At the >40% threshold, two eyes had two quadrants that met the criterion.

## 4. Discussion

In this study, we quantitatively evaluated PH lesion distribution in fellow eyes of patients with unilateral nAMD and examined its association with vortex vein asymmetry and watershed-zone status. PH lesions were more frequently distributed on the dominant side of vortex vein distribution, and eyes without a watershed zone had a significantly greater total PH lesion number. These findings suggest that PH lesion distribution is associated with vortex vein morphology and watershed-zone status.

Asymmetry in vortex vein distribution was observed in 46.6% of fellow eyes of patients with unilateral nAMD; 29.3% showed a superior-dominant pattern, and 17.2% showed an inferior-dominant pattern. Previous studies have shown that vortex vein asymmetry is more frequent in eyes with pachychoroid disease than in healthy eyes. Matsumoto et al. reported vortex vein asymmetry in 36.7% of healthy eyes and 50.0% of eyes with PNV [11]. Chung et al. reported vortex vein dilation in 30.4% of healthy eyes, 52.4% of eyes with PCV, and 40.7% of fellow eyes of patients with PCV [10]. Hiroe et al. reported vortex vein asymmetry in 38.5% of healthy eyes and 100% of eyes with CSC [9]. The frequency of vortex vein asymmetry in our study tended to be lower than that reported in eyes with pachychoroid disease and higher than that reported in healthy eyes. This intermediate frequency may reflect our evaluation of fellow eyes of patients with unilateral nAMD. However, because the assessment of vortex vein asymmetry includes a subjective component, direct comparisons between previous studies and our study should be made cautiously.

Among eyes with asymmetric vortex vein distribution, the frequency of the superior-dominant pattern did not differ significantly from that of the inferior-dominant pattern. Previous reports have shown variable patterns of vertical vortex vein predominance: Xiao et al. found that superior dominance was more frequent in both CSC and PNV [19], whereas Matsumoto et al. reported comparable frequencies of superior- and inferior-dominant patterns in both normal eyes and eyes with PNV [11]. By contrast, inferior-dominant, particularly inferotemporal-dominant, vortex vein dilation has been reported in eyes with PCV [10,20]. Pachychoroid diseases are regarded as a continuous disease spectrum that can progress from CSC to PNV and subsequently to PCV, and the frequency of vortex vein anastomoses has been reported to increase with disease progression [21]. Together, these findings raise the possibility that dominant vortex vein distribution may vary during pachychoroid disease progression. Vortex vein anastomoses may form through vascular dilation or angiogenesis as a compensatory mechanism to alleviate vortex vein congestion. Matsumoto et al. demonstrated retrograde pulsatile vortex venous flow in eyes with CSC, PNV, and PCV, showing that venous blood can flow from the dominant vortex vein into the contralateral vortex vein through intervortex venous anastomoses [22]. They proposed that retrograde pulsatile vortex venous flow may cause the contralateral vortex vein to become more dilated than the initially congested primary dominant vortex vein. If PH lesions arise from choroidal vascular hyperpermeability associated with vortex venous congestion, compensatory redistribution of venous flow over time may shift the distribution of PH lesions toward the contralateral vortex vein territory. Accordingly, eyes with extensive RPE alterations, which may reflect more chronic disease, could exhibit a different PH distribution from that observed in the present cohort. Longitudinal studies are needed to directly test this hypothesis by evaluating changes in vortex vein distribution over time.

In this study, 24.1% of eyes had no identifiable watershed zone. Previous studies reported that the proportion of eyes without a watershed zone was 20.0% in healthy eyes [11] and 31.4% in eyes with CSC [23]; thus, the proportion in our cohort of fellow eyes from patients with nAMD fell between these values. Watershed-zone disappearance is thought to result from vortex vein anastomosis formation. Accordingly, many previous studies have focused on the presence or absence of vortex vein anastomoses rather than watershed-zone status [19,22]. However, because fine vortex vein anastomoses are thought to exist in some eyes, we used watershed-zone status as an indicator of vortex vein congestion rather than directly evaluating vortex vein anastomoses. No significant association was observed between watershed-zone status and vortex vein asymmetry. Similarly, a previous study of eyes with CSC reported no association between watershed-zone status and vortex vein distribution [23]. Because vortex vein asymmetry is more frequent in eyes with pachychoroid disease than in healthy eyes, it may be involved in pachychoroid-related pathology or may reflect predisposing background factors. In contrast, vortex vein anastomosis formation and the resulting watershed-zone disappearance may reflect remodeling of choroidal drainage routes associated with vortex vein congestion.

In this study, PH lesions were more numerous on the dominant side of vortex vein distribution. Previous studies have reported that serous pigment epithelial detachments and leakage points in eyes with CSC are more frequently located on the dominant side of the vortex veins [24]. CVH also occurs more frequently on the dominant side of the vortex veins in eyes with CSC [25] and PCV [10]. In eyes with CSC, CVH has also been reported to be more frequently distributed on the dominant side of vortex veins, regardless of watershed-zone status [23]. These findings suggest that venous congestion may be more pronounced on the dominant side of vortex vein distribution and that CVH may be more likely to develop in these regions. Our finding that PH lesions were more frequently distributed on the dominant side of vortex vein distribution supports an association between PH distribution and vortex vein morphology. We also found that eyes without a watershed zone had a significantly greater total PH lesion number. A previous study of eyes with CSC reported that the number of CVH areas was significantly greater in eyes without a watershed zone [23]. Therefore, the greater PH lesion number in eyes without a watershed zone supports an association between PH formation and watershed-zone status.

In the four-quadrant analysis, the proportions of PH lesions did not differ significantly among quadrants. However, 30 eyes (51.7%) showed clustering of >50% of PH lesions within a single quadrant. These findings indicate that PH lesions are not uniformly biased toward a specific quadrant at the group level but tend to cluster locally in individual eyes. If PH lesions form in association with areas of CVH, localized clustering in different quadrants across individual eyes would be plausible because CVH distribution varies among cases. Pachydrusen have been described as a distinct drusen subtype associated with pachychoroid disease [26]. Pachydrusen are typically defined as yellow–white deposits measuring ≥125 μm, with well-defined but irregular margins, and are usually scattered or clustered throughout the posterior pole. Pachydrusen also show hyperfluorescence on late-phase ICGA [27]. Because PH and pachydrusen are both associated with pachychoroid disease, show hyperfluorescence on ICGA, and often exhibit clustered distribution, they may share overlapping pathophysiological features. In our previous studies of fellow eyes from patients with unilateral nAMD, one study classified eyes according to drusen patterns (soft drusen, subretinal drusenoid deposits, pachydrusen, and no drusen), whereas another classified eyes without drusen according to the presence or absence of PH. Eyes with pachydrusen and eyes with PH among those without drusen showed similar responses to intravitreal aflibercept therapy and comparable subfoveal choroidal thickness. These findings raise the possibility that PH and pachydrusen may represent related, but distinct, manifestations within the pachychoroid disease spectrum [15,28]. The present study also showed that total PH lesion number was associated with age. If PH lesions form in the setting of chronic vortex vein congestion and CVH, their number may increase over time as choroidal congestion persists. However, because this study was cross-sectional, temporal changes in PH lesion number could not be directly evaluated. Future longitudinal studies are needed to investigate the relationship between temporal changes in PH lesion number and choroidal hemodynamics.

This study has several limitations. First, this was a retrospective single-center study. No formal a priori sample-size calculation was performed, and the sample size was determined by the number of eligible eyes available during the study period. Second, only fellow eyes of patients with unilateral nAMD showing PH were included; therefore, the findings may not be directly generalizable to eyes with pachychoroid diseases, including CSC, PNV, and PCV. The study also could not determine whether the observed vortex vein and watershed-zone patterns were specific to PH-positive eyes or differed from normal choroidal anatomy. Third, eyes with RPE alterations larger than 0.5 disc diameter were excluded to avoid the influence of extensive mottled hyperfluorescence or hypofluorescence on PH assessment. This exclusion may have introduced selection bias, preferentially selected eyes with less extensive RPE involvement, and limited the generalizability of our findings to eyes with extensive RPE alterations. Conversely, restricting the analysis to eyes without extensive RPE alterations may have facilitated a clearer assessment of the relationships between PH distribution and dominant vortex veins or watershed-zone status, with less influence from chronic secondary changes. Hypertension was significantly more prevalent in the included cohort than in the excluded cohort. However, within the included cohort, hypertension was not significantly associated with total PH lesion number, watershed-zone status, or vortex vein distribution, suggesting that it was unlikely to fully account for the observed associations. Fourth, PH lesions were quantified using image-processing thresholds based on late-phase ICGA images; therefore, the results may have been influenced by image quality, dye leakage, or background fluorescence. Furthermore, the image-processing parameters were empirically selected and were not validated in an independent dataset. In addition, the retinal vein diameter used as an approximate internal reference was derived from only 10 eyes and may vary among individuals. Ocular magnification was also not corrected for individual axial length. These factors may have resulted in some misclassification of PH lesion size, particularly for lesions near the predefined cutoffs. Fifth, multiple secondary and exploratory analyses were conducted without formal adjustment for multiplicity; therefore, these secondary and exploratory findings should be interpreted as hypothesis-generating. Sixth, two swept-source OCT platforms were used for the qualitative assessment of vortex vein distribution and watershed-zone status. Although identical anatomical criteria were applied and segmentation boundaries were manually corrected, subtle device-related differences in image quality or en face reconstruction may have influenced these classifications. SFCT, however, was measured only using the DRI OCT -1 Atlantis. Finally, histological confirmation of PH lesions was not available; therefore, the precise anatomical substrate and formation mechanism of PH remain unclear. Future longitudinal studies with larger cohorts and multimodal imaging are needed to validate these findings and clarify the pathophysiological significance of PH.

## 5. Conclusions

PH lesions in fellow eyes of patients with unilateral nAMD were preferentially distributed on the dominant side of vortex vein distribution, and eyes without a watershed zone had a significantly greater total PH lesion number. These findings indicate that PH distribution and lesion burden are associated with vortex vein morphology and watershed-zone status and may reflect underlying alterations in choroidal venous drainage. Quantitative assessment of PH distribution may provide insight into the choroidal hemodynamics underlying pachychoroid-driven nAMD and the pathophysiological significance of PH as an imaging biomarker.

## Figures and Tables

**Table 1:** Baseline characteristics of included and excluded eyes.

Variable	Included Eyes (*n* = 58)	Excluded Eyes (*n* = 66)	Standardized Difference	Difference (95% CI)
Age (years), mean (SD)	71.8 (9.1)	72.4 (8.8)	0.07	−0.7 (−3.8–2.5)
Sex (female), No. (%)	18 (31.0)	20 (30.3)	0.02	0.7 (−15.3–16.9)
Hypertension, No. (%)	34 (58.6)	20 (30.3)	0.59	28.3 (10.8–44.1)
Diabetes, No. (%)	6 (10.3)	10 (15.2)	0.14	−4.8 (−16.5–7.4)
Smoking history (ever-smoker), No. (%)	36 (62.1)	44 (66.7)	0.10	−4.6 (−21.2–12.2)
Presence of soft drusen, No. (%)	9 (15.5)	14 (21.2)	0.15	−5.7 (−19.0–8.2)
Choroidal thickness (μm), mean (SD)	234.8 (90.0)	240.2 (96.5)	0.06	−5.4 (−38.7–27.9)

Differences were calculated as the value or proportion in included eyes minus that in excluded eyes. CI, confidence interval; SD, standard deviation.

**Table 2:** Univariable associations of clinical characteristics with total PH lesion number.

Variable	Category	*n*	Spearman’s ρ (95% CI)	*p*	Total PH Lesion Number, Median (IQR)	IRR (95% CI)	*p*
Categorical Variables							
Sex	Female	18			52.5 (16.3–66.5)	Reference	0.23
	Male	40			35.0 (19.5–71.8)	1.34 (0.83–2.17)	
Hypertension	No	24			33.0 (14.8–61.5)	Reference	0.10
	Yes	34			35.5 (22.8–74.3)	1.46 (0.93–2.28)	
Diabetes	No	52			37.0 (18.3–68.0)	Reference	0.32
	Yes	6			30.5 (21.8–66.5)	0.69 (0.33–1.43)	
Smoking history	No	22			59.5 (31.3–69.3)	Reference	0.81
	Yes	36			27.0 (18.0–65.3)	0.94 (0.60–1.49)	
Soft drusen in the fellow eye	No	49			39.0 (20.0–70.5)	Reference	0.04
	Yes	9			27.0 (11.5–59.5)	0.53 (0.29–0.97)	
Polypoidal lesion in the affected eye	No	25			35.0 (22.5–66.5)	Reference	0.29
	Yes	33			36.0 (18.0–73.0)	1.27 (0.81–2.00)	
Continuous Variables							
Age	per 1-year increase	58	0.35 (0.10–0.57)	0.008		1.04 (1.02–1.07)	0.001
SFCT	per 100-μm increase	58	0.05 (−0.19–0.29)	0.70		1.15 (0.87–1.52)	0.34

CI, confidence interval; IQR, interquartile range; IRR, incidence rate ratio; PH, punctate hyperfluorescence; SFCT, subfoveal choroidal thickness.

## Data Availability

The data used to support the findings of this study are restricted by the Kawasaki Medical School Ethics Committee to protect patient privacy. Data are available from Hiroyuki Kamao (hironeri@med.kawasaki-m.ac.jp) for researchers who meet the criteria for access to confidential data.
